# Environmental DNA (eDNA) Detection Probability Is Influenced by Seasonal Activity of Organisms

**DOI:** 10.1371/journal.pone.0165273

**Published:** 2016-10-24

**Authors:** Lesley S. de Souza, James C. Godwin, Mark A. Renshaw, Eric Larson

**Affiliations:** 1 Department of Natural Resources and Environment, University of Illinois, Champaign-Urbana, Illinois, United States of America; 2 Science and Education, The Field Museum of Natural History, Chicago, Illinois, United States of America; 3 Alabama Natural Heritage Program®, Museum of Natural History, Auburn University, Auburn, Alabama, United States of America; 4 Oceanic Institute at Hawai’i Pacific University, Shrimp Department, Waimanalo, Hawaii, United States of America; University of Hyogo, JAPAN

## Abstract

Environmental DNA (eDNA) holds great promise for conservation applications like the monitoring of invasive or imperiled species, yet this emerging technique requires ongoing testing in order to determine the contexts over which it is effective. For example, little research to date has evaluated how seasonality of organism behavior or activity may influence detection probability of eDNA. We applied eDNA to survey for two highly imperiled species endemic to the upper Black Warrior River basin in Alabama, US: the Black Warrior Waterdog (*Necturus alabamensis*) and the Flattened Musk Turtle (*Sternotherus depressus*). Importantly, these species have contrasting patterns of seasonal activity, with *N*. *alabamensis* more active in the cool season (October-April) and *S*. *depressus* more active in the warm season (May-September). We surveyed sites historically occupied by these species across cool and warm seasons over two years with replicated eDNA water samples, which were analyzed in the laboratory using species-specific quantitative PCR (qPCR) assays. We then used occupancy estimation with detection probability modeling to evaluate both the effects of landscape attributes on organism presence and season of sampling on detection probability of eDNA. Importantly, we found that season strongly affected eDNA detection probability for both species, with *N*. *alabamensis* having higher eDNA detection probabilities during the cool season and *S*. *depressus* have higher eDNA detection probabilities during the warm season. These results illustrate the influence of organismal behavior or activity on eDNA detection in the environment and identify an important role for basic natural history in designing eDNA monitoring programs.

## Introduction

The fields of conservation science and natural resource management are being transformed by a number of molecular approaches that facilitate the measurement and monitoring of biodiversity [1,2]. This includes the increasing application of environmental DNA (eDNA) to infer presence and abundance of a range of taxa in freshwater, marine, and terrestrial ecosystems [3–5]. Environmental DNA refers to DNA captured and identified from environmental samples [6–8], and has been used in a variety of conservation applications including early detection of new biological invasions or diseases (e.g., [9,10]) and monitoring the distribution of native species of conservation concern (e.g., [11,12]). Rapid growth in the application of eDNA to biodiversity monitoring and resource management may be attributable to the general tendency for this approach to be more sensitive to the detection of focal organisms relative to conventional sampling methods [13,14], as well as emerging barcoding or meta-genetics techniques that allow for characterization of entire communities from sample sites with relatively low sampling effort [15–17].

Despite the boom in use of eDNA in conservation science and natural resource management, this method still requires considerable testing and validation to define the conditions over which it does and does not work [18] as well as to advance statistical and modeling methodologies well-suited for eDNA applications [10,19]. As examples, researchers are just beginning to determine how environmental conditions influence DNA persistence times and transport distances (e.g., [20–22]), as well as how methodological choices of sample volume, storage, or laboratory processing can influence eDNA detection and quantification [23–25]. Similarly, modeling approaches with explicit corrections for detection probabilities (i.e., the rate and causes of false negatives) and the inverse issue of false positives (e.g., sample contamination) have only recently been applied to eDNA and are still not in wide use [10,19,26]. To this list of needs in refining eDNA methodologies for conservation and management, we propose as well that incorporating information on seasonal activity or behavior of focal organisms into evaluations of eDNA performance has to date been largely neglected.

Conventional field sampling methods for estimating the occupancy (presence/absence) or abundance of focal organisms have often sought to determine the influence of seasonal activity or behavior on detection probabilities, and to incorporate this information into the design of effective surveys [27,28]. For example, studies of migratory species generally opt to sample for presence or abundance over windows of time when organisms are anticipated to be present (e.g., [29,30]), whereas studies of resident species often account for how seasonally varying factors like temperature or precipitation may influence detection probability as mediated through responses of organismal activity or behavior (e.g., [31,32]). Importantly, how season or temperature affects detection probability often differs by both species and sampling methodology [33,34], yet few studies to date have considered season as a factor that may influence detection probability of eDNA (but see [13,35]). However, it is not difficult to imagine a number of ways by which seasonal patterns of temperature or precipitation and their relationships to organismal behavior or life history might affect availability of DNA in the environment–as just one example, peak timing of reproduction of focal organisms is likely to increase detectability of eDNA owing to the pulsed availability of gametes in the environment [13,36].

We report here results of a study that used eDNA to evaluate patterns of occupancy for two imperiled aquatic species of the Black Warrior River basin, Alabama, US: the Black Warrior Waterdog (*Necturus alabamensis*) and the Flattened Musk Turtle (*Sternotherus depressus*). Interestingly, these species have offset patterns of seasonal activity, with *N*. *alabamensis* active in the cool season (approximately October to April) and *S*. *depressus* active in the warm season (May to September). This fundamental natural history difference between two sympatric species of high conservation need allowed us to make a novel evaluation of the potential effect of season and associated behavior of focal organisms on detection probability of eDNA. While our findings have direct implications for the conservation and management of these two narrowly endemic, cryptic species, we also provide a more general evaluation of whether eDNA as a sampling methodology is either robust or sensitive to variation in activity or behavior of focal organism across seasons, which can guide sampling design for other taxa when similar natural history information is available.

## Methods

### Study System

The southeastern US is a hotspot of temperate freshwater biodiversity and high percentages of aquatic species in this region are threatened with extinction, including reptiles and especially wholly aquatic salamanders [37–39]. Emblematic of freshwater conservation challenges in the southeastern US are the aquatic salamander *N*. *alabamensis* and turtle *S*. *depressus*, two endemics with overlapping ranges in the upper Black Warrior River basin of Alabama, US. Both species prefer clear, rocky, permanent streams and rivers [40–42], and are believed to have suffered population declines due to habitat fragmentation and degradation in water quality associated with land use change (e.g., logging, mining, urbanization, etc.). Recent surveys report *N*. *alabamensis* and *S*. *depressus* from only 14 and 60 localities, respectively, constituting over a 50% loss in the historic range of each species [40,43,44]. The Alabama Department of Conservation and Natural Resources recognizes both *N*. *alabamensis* and *S*. *depressus* on its state protected species list, whereas the US Fish and Wildlife Service has listed *S*. *depressus* as threatened under the US Endangered Species Act and *N*. *alabamensis* is currently under review for listing [45]

Conventional field sampling approaches used for one or both of these species have included dip netting, baited trapping, and backpack electrofishing, but these methods are labor-intensive with generally low rates of detection for *N*. *alabamensis* and *S*. *depressus*. Accordingly, eDNA offers a promising alternative for surveys of *N*. *alabamensis* and *S*. *depressus* given its past successful application to similar species [11,13]. However, *N*. *alabamensis* is a cool-season active species that reproduces over the winter, whereas *S*. *depressus* reduces activity over the winter and is instead generally most active during the warm season [42, 44]. As such, these species seemed well-suited to evaluate the sensitivity of eDNA to season of the year and related activity levels of focal organisms. Does eDNA detect presence of a species irrespective of season and behavior, or does this method perform better (e.g., achieve higher detection probabilities) in seasons when focal organisms are more active?

### Field Sampling

**Fig 1:**
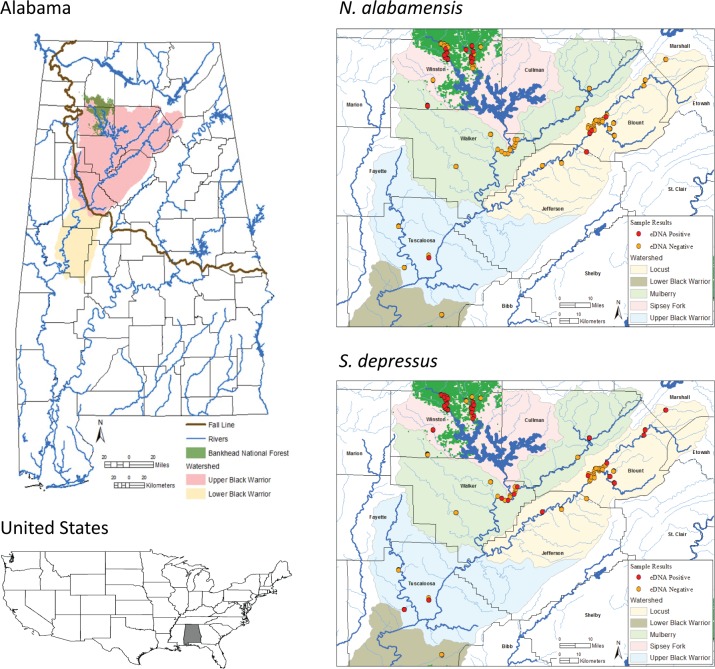
Location of environmental DNA (eDNA) sample sites and observed detections (negative/positive) for the Black Warrior Waterdog (*Necturus alabamensis*) and Flattened Musk Turtle (*Sternotherus depressus*) in the Black Warrior River drainage, Alabama, United States. Major Black Warrior watersheds above the Fall Line (see main text) are noted along with counties of Alabama and the location of the Bankhead National Forest.

Our study was conducted in 2013 and 2014 at eDNA sample sites across the upper Black Warrior River including all major tributary watersheds above the Fall Line (i.e., the transition between higher gradient upland and lower gradient coastal plain streams in the southeastern US; Fig 1). Sample sites were selected from known historic localities of one or both species. In 2013, we sampled 22 sites at road crossings of streams or rivers, with 12 of these sites sampled in both the cool and warm season, 4 sites sampled only in the cool season, and 6 sites sampled only in the warm season. For 2013, cool season sampling occurred between January 23^rd^ and March 7^th^, whereas warm season sampling occurred between April 23^rd^ and August 14^th^. We refer to the warm season as beginning in May throughout the manuscript for simplicity, because only a total of three warm season samples across the entire study were collected at the end of April 2013.

In 2014, we sampled eight continuous stream reaches by canoe, with eDNA sample sites at evenly spaced intervals over the length of each float, although separated by an average distance of approximately 1 km. An emerging consensus of studies suggests that eDNA will not generally be transported over 1 km in flowing waters [46], although there may be exceptions [21]. The location, sample dates, and number of sample sites for eDNA collected from these continuous stream reaches in summer of 2014 are provided in S1 Table. Altogether, we collected eDNA at 16 cool season and 18 warm season sample sites in 2013, and 60 cool season and 48 warm season sample sites in 2014, for a total of 142 sample sites by season and year combinations.

At each site, we collected three replicate water samples each in a 1 L Nalgene bottle that had been previously decontaminated with a 30% bleach rinse followed by two distilled water rinses. We also used a single 1 L Nalgene bottle of distilled water at each site as a cooler blank or control. Replicate water samples and their cooler blank from each site were placed in zip lock bags and stored for no more than eight hours prior to vacuum filtering of each 1 L volume through 0.80 μm cellulose nitrate filters. Filters were then stored in vials of cetyl trimethylammonium bromide (CTAB) buffer for transport back to the laboratory, where they were subsequently stored in a -20 C freezer prior to DNA extraction. For more detail on the performance of CTAB storage of eDNA filter samples, see [23].

### Laboratory Methods

Primers for *N*. *alabamensis* and *S*. *depressus* were designed using PrimerHunter [47] and checked for cross-amplification with closely related taxa (S2 Table). Due to the paucity of publicly available sequence data for both the focal *Necturus* and *Sternotherus* taxa, fragments from three mitochondrial genes were Sanger sequenced for taxa of interest. Primer design maximized potential mismatches between targeted taxa and co-occurring non-target taxa while ensuring diagnostic site differences between species in the intervening regions (between forward and reverse primers), and the optimal sets of primers for *Necturus depressus* and *Sternotherus depressus* were chosen based on the increased inefficiency of amplification for non-targeted taxa (largest difference in Cq between target and non-target taxa). We chose a primer pair for *N*. *alabamensis* that produced a 124bp amplicon, and a primer pair for *S*. *depressus* that produced a 130bp amplicon:

Nalabamensis_COI_4F (5’-CGTATTAATTACAGCCA-3’)

Nalabamensis_COI_4R (5’-CGGGTCACCTCCTCC-3’)

Sdepressus_16S_2F (5’-TTCAAATATCCATCAACTAGAAACAA-3’)

Sdepressus_16S_2R (5’-GGTGTAGAATTTATGTTCTGTCTTCG-3’)

We extracted DNA using a modified CI extraction and isopropanol precipitation that has been found to produce higher yields of eDNA than some other methods [24]. One extraction blank was included for every 23 samples being extracted for DNA to insert another stage where contamination can be monitored. We then ran real-time qPCR assays of our eDNA extractions using six replicates of each sample in the following 20 μl mixes: 6.25 μl of sterile water, 4 μl of 5X GoTaq® Flexi Buffer (Promega), 0.4 μl of 10 mM dNTPs, 1.6 μl of 25 mM MgCl_2_, 0.3 μl of each 10 μM primer, 0.15 μl of GoTaq® Flexi DNA Polymerase, 1 μl of 20X EvaGreen in water (Biotium), 2 μl of 4 μg/μl Bovine Serum Albumin (BSA), and 4 μl of DNA template. Mastercycler® ep *realplex* (Eppendorf) cycling conditions were as follows: an initial denaturation at 95 C for 3 minutes; 40 cycles of denaturation at 95 C for 30 seconds, annealing at 55 C (*N*. *alabamensis*) or 62 C (*S*. *depressus)* for 45 seconds, and extension at 72 C for 1 minute; followed by a melting curve analysis transitioning from 60 to 95 C over 20 minutes.

Each real-time qPCR assay plate also included two negative controls (sterile water in place of template DNA) and a single positive control (tissue-derived genomic DNA extraction at 1 pg/μl concentration). Positive amplifications were further evaluated for the expected melting curve. A single replicate from each putative positive was submitted for unidirectional Sanger sequencing with the reverse primer to confirm as either *N*. *alabamensis* or *S*. *depressus*. Priming regions were trimmed from resulting sequences in consideration of the eDNA sequence match to the targeted species’ sequence. It is important to note that amplification of tissue-derived DNA from non-target taxa was observed for both the *N*. *alabamensis* and *S*. *depressus* primer sets and as such, Sanger sequence confirmation of all eDNA amplifications took advantage of diagnostic differences imbedded in the intervening regions for both assays.

Our eDNA field samples were only considered positive for either focal species if they demonstrated all of the following: (1) positive and negative controls on same 96-well plate performed as expected, (2) eDNA sample displayed expected amplification curve, (3) eDNA sample displayed expected melting curve peak, and (4) Sanger sequencing produced a clean chromatogram that matched the expected sequence for *N*. *alabamensis* or *S*. *depressus*. For each eDNA sample that met all of these criteria, the corresponding field and lab contamination blanks (cooler and extraction) were processed to verify that the results were not due to contamination during sample collection or processing. If any of these contamination blanks were positive under the previously listed criteria, the eDNA sample was removed from further consideration.

### Statistical Analysis

We modeled occupancy and detection probability for *N*. *alabamensis* and *S*. *depressus* eDNA using hierarchical linear models [27], where the three replicate water samples per eDNA sample site and season combination served as repeated measures for estimation of eDNA detection probability [10]. Relating occupancy patterns of *N*. *alabamensis* and *S*. *depressus*, as inferred by eDNA, to a series of abiotic or land cover predictors was not necessarily the primary focus of this study, owing in part to our non-random sampling concentrated on historic occurrence localities for both species. However, this analysis is retained here in order to identify some environmental factors associated with persistence of these species at present, with the caveat that our sampling omits environmental conditions where these species were never historically observed. Conversely, hierarchical occupancy estimation models are well-suited to use replicated eDNA samples to model factors affecting detection probability for this tool [10], and as such our primary emphasis is on relating season of eDNA sampling to detection probability as a consequence of patterns of focal organism behavior or activity.

We selected six Geographic Information System (GIS)-derived abiotic and land cover predictors to model patterns of eDNA occupancy for *N*. *alabamensis* and *S*. *depressus* at three scales, from point of sample site location to the entire upstream watershed (Table 1). These occupancy predictors were derived from national land cover, soils, and hydrography datasets for the US as extracted from the geographic coordinates of our sample locations by an automated online tool (GeoData Crawler, www.geodatacrawler.com, accessed 8 December 2015; [48]). We expected that terrain slope at the point of our sample site locations might explain occupancy for both species owing to their habitat preferences for rocky stream substrates that are most likely to be associated with higher gradient, erosional stream reaches as opposed to lower gradient, depositional stream reaches. At a local scale consisting of a 100 m diameter riparian buffer extending upstream within a 300 m radius of each sample site location, we included both percent forest canopy cover and average soil depth to rock as predictors. Forest canopy cover was included as a measure of habitat intactness or anthropogenic disturbance in this historically forested region, whereas depth to rock was included as a measure of prospective availability of preferred rock habitat in stream channels. At a predictor scale consisting of the entire upstream watershed from each sample site location, we included both percent forest canopy cover and percent impervious surface cover, again as measures of anthropogenic impact or disturbance on the landscape. Impervious surface was omitted at the local scale owing to very low impervious surface values in immediate proximity to our sample points. We also included watershed area itself as a predictor to represent a habitat gradient from small headwater streams to larger main channel rivers; related predictors like Strahler or Shreve stream order were omitted owing to high correlations (r > 0.80) with watershed area. Point, local, and watershed predictors were included both to represent potential effects of different scales on occupancy of both species [49], as well as how inference of focal organism occupancy at a particular sample site location might also reflect upstream conditions in the watershed owing to eDNA transport [21,46].

**Table 1:** Predictor variables for occupancy estimation using environmental DNA (eDNA) detections of the Black Warrior Waterdog (*Necturus alabamensis*) and Flattened Musk Turtle (*Sternotherus depressus*) given at three spatial scales: point, local riparian (100 m riparian buffer upstream and within 300 m radius of point), and watershed (entire upstream area from point). Predictor variables are given with abbreviations for reference in subsequent tables, brief descriptions with units, data sources with references, and the mean and range.

Scale				
Variable (Abbreviation)		Description (Unit)	Data Source	Mean (Range)
Point				
	Slope (pslope)	Terrain slope (°)	National Hydrography Dataset Plus (USEPA and USGS, 2012)	5.7 (0.1–27.7)
Local riparian				
	Forest canopy (lcnpy)	Forest cover (%)	National Land Cover Database 2006 (Homer et al., 2007; Fry et al. 2011)	61.6 (9.7–90.0)
	Rock depth (lrockd)	Soil thickness (m)	United States General Soils Map (USDA, 2006)	0.87 (0.70–1.52)
Watershed				
	Forest canopy (wcnpy)	Forest cover (%)	National Land Cover Dataset 2006 (Homer et al., 2007; Fry et al. 2011)	61.6 (9.7–90.0)
	Impervious surface (wimpv)	Impervious surface (%)	National Land Cover Dataset 2006 (Homer et al., 2007; Fry et al. 2011)	0.9 (0.1–6.2)
	Watershed area (wwshd)	Watershed area (km^2^)	National Hydrography Dataset Plus (USEPA and USGS, 2012)	958.3 (1.6–4133.2)

Detection probability of *N*. *alabamensis* and *S*. *depressus* eDNA was modeled with two covariates, year and season of sample. We included year to account for potential differences in abiotic conditions between 2013 and 2014, as well as differences in the eDNA sampling protocol between these years (individual point vs. continuous river reaches). Inclusion of year as a predictor for detection probability but not occupancy assumes a closed occupancy state for both species (i.e., neither species was extirpated from or colonized sample sites between years). Season was modeled as either cool (January-April; but see above) or warm (May-August) season categories at time of sample collection, and also assumes that season affects detection probability of these organisms through activity levels or mating behavior rather than through immigration or emigration between sites. However, we believe these assumptions are reasonable given that both species are relatively long-lived and unlikely to have been extirpated from sites over a single year, and also exhibit small home ranges without evidence of intra- or inter-annual migratory behavior over long distances [50,51].

The hierarchical linear models employed here are based on the dependency of the observed occupancy state (ψ) on the ability to detect focal organisms when actually present (detection probability, *P;* [27]). Detection probability is inferred through repeated sampling in space or time; for eDNA sampling, this may often mean use of multiple replicated water samples at a site as units for estimating eDNA detection probability [10]. We modeled occupancy and detection probability using the *unmarked* library in version 3.1.2 of the statistical program R [52] based on the two-level occupancy model of Mackenzie et al. [27]. Comparisons between candidate models including combinations of occupancy and detection probability predictors were made using Akaike’s Information Criterion (AIC), where models within ΔAIC<2 of the single most supported model were considered equivalent [53]. We also characterized the relationship between replication level of eDNA water samples per site (n) and detection probability using McArdle’s [54] cumulative detection probability equation based on estimates of our single most supported model.

## Results

### eDNA Detections

We detected *N*. *alabamensis* eDNA at two individual sites (two total water samples) from the 2013 sampling and six of the stream reaches (24 total water samples) from the 2014 continuous sampling. These included new localities or distributional records for *N*. *alabamensis*, as inferred by eDNA, at Gurley Creek in Jefferson County, Locust Fork in Blount County, Sipsey Fork and Rush and Brushy creeks in the Bankhead National Forest (Fig 1). Twenty-one water samples where *N*. *alabamensis* was detected by eDNA were collected in the cool season, whereas only five were collected in the warm season. We detected *S*. *depressus* eDNA at nine individual sites from the 2013 sampling (10 total water samples) and six of the stream reaches (34 total water samples) from the 2014 continuous sampling. Sites of eDNA detection for *S*. *depressus* included Slab Creek in Marshall County, Mulberry Fork at the confluence with the Duck River in Cullman County, and Carroll and Yellow creeks in Tuscaloosa County (Fig 1). Twenty-nine water samples where *S*. *depressus* was detected by eDNA were collected in the warm season, and 15 were collected in the cool season. Sanger sequencing confirmed positive eDNA detections by qPCR for both of our focal species, and there were no qPCR amplifications of *N*. *alabamensis* and *S*. *depressus* DNA in any field or laboratory negative controls. Accordingly, we have no evidence that any of our results were the consequence of contamination during water sample collection, filtering, laboratory processing, or qPCR assays.

### Occupancy Estimation

Four models for *N*. *alabamensis* and seven models for *S*. *depressus* were within ΔAIC≤2 of the most supported model and consequently were considered equivalent (Table 2). Percent cover of impervious surface at the watershed scale was included in all of these models for *N*. *alabamensis*, which consistently had a negative occupancy relationship to this measure of anthropogenic land use intensity (Table 3). Occupancy of *N*. *alabamensis* also had a positive relationship with steeper terrain slopes as initially hypothesized, and a negative relationship with watershed area, indicating persistence of this species primarily in smaller watersheds or streams. Conversely, *N*. *alabamensis* had a positive relationship with soil depth to rock, contradicting our expectation of preference for this species for rockier stream substrates. Similar to *N*. *alabamensis*, *S*. *depressus* occupancy had a positive relationship with terrain or stream gradient (occurring in steeper streams), a negative relationship with watershed area (occurring in smaller watersheds or streams), and a negative relationship with percent impervious surface cover at the watershed scale. We found no consistent relationship of soil depth to rock on occupancy patterns for *S*. *depressus*, and neither species showed a consistent relationship to forest canopy cover at either the local or watershed scales (Table 3).

**Table 2:** Specifications for occupancy (Ψ) and detection probability (*P*) modeling using environmental DNA (eDNA) for the Black Warrior Waterdog (*Necturus alabamensis*) and Flattened Musk Turtle (*Sternotherus depressus*), with ΔAIC and model weights (*w*) for the ten most supported models and all other considered models combined. Models with ΔAIC ≤2 are denoted with *. Predictor abbreviations are given in Table 1.

*N*. *alabamensis*			*S*. *depressus*		
Model	ΔAIC	*w*	Model	ΔAIC	*w*
1. Ψ(wimpv), *P*(season)*	0	0.29	1. Ψ(pslope, wwshd), *P*(season)*	0	0.12
2. Ψ(pslope, wimpv), *P*(season)*	1.54	0.14	2. Ψ(wwshd), *P*(season)*	0.42	0.10
3. Ψ(wimpv, wwshd), *P*(season)*	1.77	0.12	3. Ψ(pslope), *P*(season)*	0.47	0.10
4. Ψ(lrockd, wimpv), *P*(season)*	1.96	0.11	4. Ψ(pslope, wimpv), *P*(season)*	0.61	0.09
5. Ψ(wimpv), *P*(season, year)	2.70	0.08	5. Ψ(wimpv), *P*(season)*	0.98	0.07
6. Ψ(pslope, wimpv, wwshd), *P*(season)	3.81	0.04	6. Ψ(wimpv, wwshd), *P*(season)*	1.54	0.06
7. Ψ(wimpv), wwshd, *P*(season, year)	3.85	0.04	7. Ψ(pslope, wimpv, wwshed), *P*(season)*	1.65	0.05
8. Ψ(lrockd, wimpv), *P*(season, year)	4.43	0.03	8. Ψ(lrockd, wimpv, wwshed), *P*(season)	2.12	0.04
9. Ψ(pslope, wimpv), *P*(season, year)	4.59	0.03	9. Ψ(lrockd, wwshed), *P*(season)	2.28	0.04
10. Ψ(wimpv), *P*(.)	4.67	0.03	10. Ψ(lrockd, pslope), *P*(season)	2.33	0.04
All other models (30)	≥5.65	0.09	All other models (27)	≥2.63	0.29

**Table 3:** Coefficients for most support models (ΔAIC ≤2) of occupancy (Ψ) and detection probability (*P*) using environmental DNA (eDNA) for the Black Warrior Waterdog (*Necturus alabamensis*) and Flattened Musk Turtle (*Sternotherus* depressus; Table 2). Predictor abbreviations are given in Table 1.

	Occupancy (Ψ)					Detection Probability (*P*)	
Model (Table 2)	Intercept (SE)	lrockd (SE)	pslope (SE)	wimpv (SE)	wwshd (SE)	Intercept (SE)	season (SE)
*N*. *alabamensis*						-1.06 (0.37)	-1.28 (0.50)
1.	1.17 (0.93)			-1.79 (0.61)		-1.00 (0.36)	-1.31 (0.50)
2.	0.73 (0.96)		0.04 (0.06)	-1.63 (0.59)		-1.14 (0.48)	-1.30 (0.49)
3.	1.64 (1.47)			-1.80 (1.09)	-0.0003 (0.0008)	-1.09 (0.48)	-1.28 (0.49)
4.	0.43 (5.48)	0.03 (0.19)		-1.86 (0.97)			
*S*. *depressus*							
1.	-0.21 (0.45)		0.07 (0.05)		-0.0004 (0.0004)	-1.38 (0.35)	1.74 (0.40)
2	0.35 (0.35)				-0.0006 (0.0004)	-1.50 (0.34)	1.89 (0.40)
3.	-0.66 (0.36)		0.10 (0.06)			-1.45 (0.34)	1.75 (0.39)
4.	-0.22 (0.48)		0.07 (0.06)	-0.38 (0.29)		-1.43 (0.34)	1.74 (0.40)
5.	0.26 (0.37)			-0.51 (0.29)		-1.37 (0.34)	1.69 (0.40)
6.	0.65 (0.42)			-0.22 (0.26)	-0.0006 (0.0004)	-1.32 (0.34)	1.68 (0.40)
7.	0.10 (0.52)		0.05 (0.05)	-0.14 (0.26)	-0.0005 (0.0004)	-1.32 (0.35)	1.68 (0.40)

### Detection Probability

**Fig 2:**
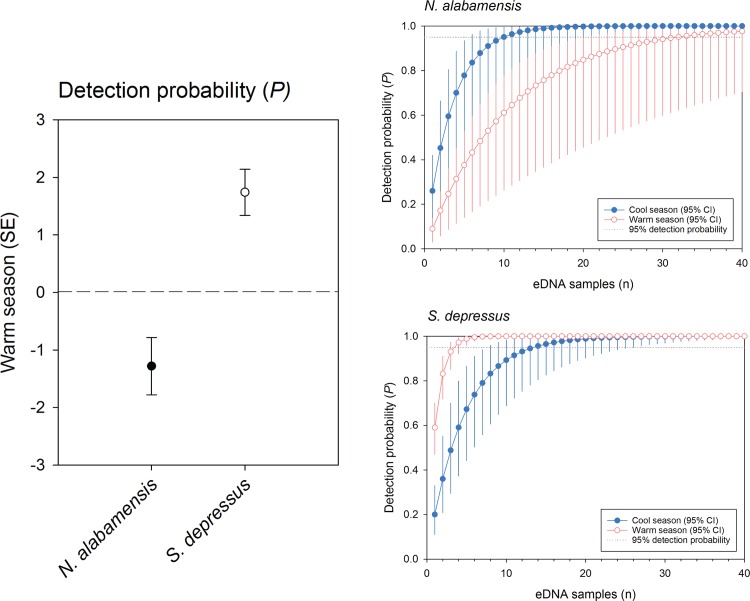
Effect of season on detection probability of environmental DNA (eDNA) for the Black Warrior Waterdog (*Necturus alabamensis*) and Flattened Musk Turtle *Sternotherus depressus* based on covariates (with standard error) from the most supported model for each species (Table 2 and Table 3), and cumulative detection probabilities for each species by number of eDNA replicates (n) per sample site for both cool and warm seasons with 95% confidence intervals. 95% detection probability is denoted by a gray dashed line.

All equivalent, most supported models for both *N*. *alabamensis* and *S*. *depressus* included season as a predictor for detection probability, and none included year (Table 2). Detection probability for *S*. *depressus* eDNA had a positive relationship with the warm season, whereas detection probability for *N*. *alabamensis* eDNA had a negative relationship with the warm season (Table 3; Fig 2). Cumulative detection probabilities per sample replication (n) at a site for both species based on estimates from single most supported models further illustrate strong effects of season on eDNA detection probability for our focal organisms (Fig 2). Overall eDNA detection probabilities were higher for *S*. *depressus*, which we estimated would require only four eDNA replicate water samples at an occupied site for a 95% probability of detection in the warm season, and 14 replicate water samples for a 95% probability of detection in the cool season. Conversely, we estimated that 10 eDNA replicate water samples at an occupied site would be necessary for 95% detection probability of *N*. *alabamensis* in the cool season, and up to 32 replicates would be necessary for 95% probability of detection for this species at occupied sites in the warm season (Fig 2).

## Discussion

Our application of species-specific eDNA assays detected both of our focal organisms across their extant ranges, and allowed us to infer patterns influencing their present-day occupancy. In particular, we found that presence of both *N*. *alabamensis* and *S*. *depressus* was negatively related to area of impervious surface at the watershed scale, and that both species were also more likely to occur in the high-gradient headwater streams of smaller watersheds. Importantly, we also found that eDNA detection probability for both species was strongly affected by season of sampling, with eDNA detection probability highest for *N*. *alabamensis* in the cool season and *S*. *depressus* in the warm season, consistent with the known natural history of both species. This finding illustrates the value of incorporating natural history knowledge into the design of eDNA sampling programs, and emphasizes that this technique shares with many conventional sampling approaches a dependence on seasonality of organism behavior or activity.

Our occupancy results suggest that both *N*. *alabamensis* and *S*. *depressus* primarily persist in smaller, high gradient headwater streams with low anthropogenic impacts. However, we did not find expected relationships between occupancy of both species and shallower soil depths to rocky substrates, which might suggest that this interpolated GIS variable is not necessarily a good proxy for in-stream habitat conditions used by our focal organisms. In addition, a large number of occurrences for both species were found in the two major headwater streams of the Bankhead National Forest, identifying this federally-owned and managed property as an important conservation area for *N*. *alabamensis* and *S*. *depressus*. Environmental DNA detections expanded the known range of both species to a number of new locations, whereas failure to detect eDNA at some historic localities may represent range declines for *N*. *alabamensis* and *S*. *depressus* [43,55]. However, we note that our estimates of cumulative eDNA detection probabilities for both species across warm and cool seasons provide guidance on the level of replication necessary in future monitoring to determine whether non-detections represent actual absences or instead false negatives. *Necturus alabamensis* in particular would likely benefit from a higher level of sample replication within site locations even under cool season conditions, whereas our level of warm season sample replication for *S*. *depressus* was likely adequate to detect presence of this species with high confidence. As such, our study provides guidance on sample design and effort for eDNA monitoring of both *N*. *alabamensis* and *S*. *depressus* in the future, as well as similar organisms in Alabama and other regions. For example, 52% of freshwater turtles native to North America are found in Alabama with 43% requiring conservation attention [37,56,57], and our study is notably among the first successful applications of eDNA to monitor populations and distributions of aquatic turtles (but see [58]).

Few studies to date have considered season of eDNA sampling in evaluations of the effectiveness of this tool. As one related exception, Spear et al. [13] found that abundance of eDNA for the Eastern Hellbender (*Cryptobranchus alleganiensis*) was highest in the autumn breeding season for this large aquatic salamander relative to the summer, presumably owing to active combat between competing males and release of gametes. Laramie et al. [35] similarly reported that eDNA concentrations for Chinook Salmon (*Oncorhychnus tshawytscha*) peaked during the spawning run of these fish, and spawn timing has also been observed to affect abundance of eDNA for invasive Bigheaded Carp species (*Hypophthalmichthys* spp.) in the Mississippi River basin [36]. Yet the majority of eDNA studies neglect season or sample timing as a consideration in performance of this tool, whether for single species assays like our study or as a mechanism explaining the variable performance of eDNA for some taxa in emerging meta-genetics or meta-bacording approaches [15,17,19]. Our comparison of eDNA detection probabilities for contrasting cool and warm season active herptiles reveals the potential importance of seasonality of focal organism behavior on designing and interpreting field studies using this tool. Specifically, *N*. *alabamensis* are consistently observed over winter or cool season months with swollen cloaca indicating reproductive activity, which involves internal fertilization and release of egg masses on the underside of rocks [51]. We believe that the cool season reproductive behavior of *N*. *alabamensis* increases eDNA detection probability for this species as per Spear et al. [13], whereas *S*. *depressus* instead reduces activity through the cool season and is more active for both foraging and reproductive behavior in late spring and summer [42,44,50], when we observed highest eDNA detection probabilities for this aquatic turtle.

Emerging molecular approaches like eDNA have clear, powerful implications for advancing the fields of conservation science and natural resource management [6,8], yet our study demonstrates that these tools are not invulnerable to the constraints that organismal life history or natural history impose on effective field sampling design [32,32]. We propose that like conventional sampling approaches, eDNA will be most effective when sample timing is synched to peak abundance of migratory species or seasonal activity levels of resident species. *A priori* knowledge of these phenological patterns of organism presence, abundance, and activity remains dependent on a robust role for natural history in the fields of ecology, evolution, and conservation, and further justifies efforts to reverse declines of this foundational field of knowledge [59]. We conclude by emphasizing that not all improvements in eDNA methodology will necessarily arise from technological advancements, but instead propose that some will arise from better integrating good field biology, natural history knowledge, and sample design into applications of this tool.

## Supporting Information

S1 Table Sampling sites on stream reaches. (PDF)

S2 Table Primer design and testing. (PDF)
