# Supplementary material for: Environmental DNA (eDNA) Detection Probability Is Influenced by Seasonal Activity of Organisms
Source: PLoS One. 2016 Oct 24;11(10):e0165273. doi: 10.1371/journal.pone.0165273 (PMC5077074; doi:10.1371/journal.pone.0165273)
Supplement: S1 Table — (PDF) [file pone.0165273.s001.pdf]

de Souza LS, Godwin JC, Renshaw MA, Larson ER. 2016. Environmental DNA (eDNA) Detection Probability is Influenced by Seasonal Activity of Organisms. Plos One.

S1 Table. The location, cool or warm season sampling dates, and number of eDNA sample sites used during 2014 canoe floats of stream reaches in Alabama, US.

**Stream reach**	**County**	**Cool (# Samples)**	**Warm (# Samples)**
Locust Fork between AL 160 & Co. 13	Blount	20 MAR (10)	15 JUL (10)
Locust Fork between Co. 13 & Armstrong-Center Rd	Blount	2 APR (10)	17 JUL (8)
Mulberry Fork	Walker	27 JAN (5)	10 JUN (5)
Warrior River	Walker	27 JAN (5)	10 JUN (5)
Brushy Creek between NFR 255 & US 278	Winston	4 MAR (10)	24 JUN (10)
Sipsey Fork below AL 33	Winston	28 JAN (5)	
Sipsey Fork at NFR 1000	Winston	19 FEB (5)	
Sipsey Fork between Co. 60 & AL 33	Winston	26 FEB (10)	7 AUG (10)
