# Supplementary material for: Environmental DNA (eDNA) Detection Probability Is Influenced by Seasonal Activity of Organisms
Source: PLoS One. 2016 Oct 24;11(10):e0165273. doi: 10.1371/journal.pone.0165273 (PMC5077074; doi:10.1371/journal.pone.0165273)
Supplement: S2 Table — (PDF) [file pone.0165273.s002.pdf]

de Souza LS, Godwin JC, Renshaw MA, Larson ER. 2016. Environmental DNA (eDNA) Detection Probability is Influenced by Seasonal Activity of Organisms. Plos One.

S2 Table. Primer design and testing.

Necturus alabamensis

Fragments were PCR amplified and Sanger sequenced for *Necturus alabamensis*, *N. lodingi*, *N. punctatus*, and *N. maculosus* from three mitochondrial genes: the 16S ribosomal RNA gene (16SA-L and 16SB-H; Vences *et al.* 2005; 551bp fragment), the *cytb* gene (H15149 and L14735; Burgener and Hubner 1998; 413bp fragment), and the cytochrome c oxidase I gene (LEP-F1 and LEP-R1; Hebert *et al.* 2004; 658bp fragment). PrimerHunter [47] was utilized for primer design with sequences from *Necturus alabamensis* as the targets and sequences from *N. lodingi*, *N. punctatus*, N. *maculosus*, and *N. beyeri* (GenBank NC_023341) as the nontargets. Primers were synthesized by Integrated DNA Technologies and tested for amplification with *Necturus alabamensis* DNA, extracted from both tissue and filtered mesocosm water, as well as reduced amplification with tissue-derived DNA from *N. lodingi* and *N. maculosus*. The best primer pair, Nalabamensis_COI_4F (5'-CGTATTAATTACAGCCA-3') and Nalabamensis_COI_4R (5'-CGGGTCACCTCCTCC-3'), produced a 124bp amplicon.

Table A1- Specificity of priming sites to *Necturus* species evaluated in the current study.

		Forward																		Reverse															
	Sample	C	G	T	A	T	T	A	A	T	T	A	C	A	G	C	C	A		C	G	G	G	T	C	A	C	C	T	C	C	T	C	C	Specificity
Necturus alabamensis	1	.	.	.	.	.	.	.	.	.	.	.	.	.	.	.	.	.		.	.	.	.	.	.	.	.	.	.	.	.	.	.	.	100%
	2	.	.	.	.	.	.	.	.	.	.	.	.	.	.	.	.	.		.	.	.	.	.	.	.	.	.	.	.	.	.	.	.	100%
	3	.	.	.	.	.	.	.	.	.	.	.	.	.	.	.	.	.		.	.	.	.	.	.	.	.	.	.	.	.	.	.	.	100%
	4	.	.	.	.	.	.	.	.	.	.	.	.	.	.	.	.	.		.	.	.	.	.	.	.	.	.	.	.	.	.	.	.	100%
Necturus lodingi	1	.	.	.	.	.	.	.	.	.	.	.	.	.	.	.	T	.		.	.	.	.	.	.	G	.	.	.	.	.	G	.	.	91%
	2	.	.	.	.	.	.	.	.	.	.	.	.	.	.	.	T	.		.	.	.	.	.	.	G	.	.	.	.	.	G	.	.	91%
	3	.	.	.	.	.	.	.	.	.	.	.	.	.	.	.	T	.		.	.	.	.	.	.	G	.	.	.	.	.	G	.	.	91%
	4	.	.	.	.	.	.	.	.	.	.	.	.	.	.	.	T	.		.	.	.	.	.	.	G	.	.	.	.	.	G	.	.	91%
Necturus maculosus	1	.	.	.	.	.	.	.	.	.	.	.	.	.	.	.	T	.		.	.	.	.	.	.	G	.	.	.	.	.	G	.	.	91%
	2	.	.	.	.	.	.	.	.	.	.	.	.	.	.	.	T	.		.	.	.	.	.	.	G	.	.	.	.	.	G	.	.	91%
Necturus beyeri	NC_023341	.	.	.	.	.	.	.	.	.	.	.	.	.	.	.	T	.		.	.	.	.	.	.	G	.	.	.	.	.	G	.	.	91%
Necturus punctatus	1	A	.	.	.	.	.	.	.	.	C	.	.	.	.	.	A	.		T	.	.	A	.	.	C	.	.	C	.	.	.	.	.	78%

Real-time PCR assays of extractions (tissue-derived and filtered mesocosm water) were run with three replicates of each sample in the following 20µl mixes: 4.85µl of sterile water, 4µl of 5X GoTaq® Flexi Buffer (Promega), 0.4µl of 10mM dNTPs, 1.6µl of 25mM $MgCl_2$, 1µl of each 10µM primer, 0.15µl of GoTaq® Flexi DNA Polymerase, 1µl of 20X EvaGreen in water (Biotium), 2µl of 4µg/µl Bovine Serum Albumin (BSA), and 4µl of DNA extract. Mastercycler® ep *realplex* (Eppendorf) cycling conditions were as follows: an initial denaturation at 95°Celsius for 3 minutes; 40 cycles of denaturation at 95°Celsius for 30 seconds, annealing at 55°Celsius for 45 seconds, and extension at 72°Celsius for 1 minute; followed by a melting curve analysis transitioning from 60° to 95°Celsius over 20 minutes.

Sternotherus depressus

Fragments were PCR amplified and Sanger sequenced for *Sternotherus depressus*, *S. odoratus*, *S. minor minor*, and *S. minor peltifer* from three mitochondrial genes: the 16S ribosomal RNA gene (16SA-L and 16SB-H; Vences *et al.* 2005; 553bp fragment), the *cytb* gene (H15149 and L14735; Burgener and Hubner 1998; 415bp fragment), and the cytochrome c oxidase I gene (LEP-F1 and LEP-R1; Hebert *et al.* 2004; 658bp fragment). PrimerHunter [47] was utilized for primer design with sequences from *Sternotherus depressus* as the targets and sequences from *S. odoratus*, *S. carinatus* (GenBank NC_017607), *S. minor minor*, and *S. minor peltifer* as the nontargets. Primers were synthesized by Integrated DNA Technologies and tested for amplification with *Sternotherus depressus* DNA, extracted from both tissue and filtered mesocosm water, as well as reduced amplification with tissue-derived DNA from *S. odoratus*, *S. minor minor*, and *S. minor peltifer*. The best primer pair, Sdepressus_16S_2F (5'-TTCAAATATCCATCAACTAGAAACAA-3') and Sdepressus_16S_2R (5'-GGTGTAGAATTTATGTTCTGTCTTCG-3'), produced a 130bp amplicon.

Table A2- Specificity of priming sites to *Sternotherus* species evaluated in the current study.

		Forward																									Reverse																									
	Sample	T	T	C	A	A	A	T	A	T	C	C	A	T	C	A	A	C	T	A	G	A	A	A	C	A	A	G	G	T	G	T	A	G	A	A	T	T	T	A	T	G	T	T	C	T	G	T	C	T	T	C	G	Specificity
Sternotherus depressus	1	.	.	.	.	.	.	.	.	.	.	.	.	.	.	.	.	.	.	.	.	.	.	.	.	.	.	.	.	.	.	.	.	.	.	.	.	.	.	.	.	.	.	.	.	.	.	.	.	.	.	.	.	100%
	2	.	.	.	.	.	.	.	.	.	.	.	.	.	.	.	.	.	.	.	.	.	.	.	.	.	.	.	.	.	.	.	.	.	.	.	.	.	.	.	.	.	.	.	.	.	.	.	.	.	.	.	.	100%
	3	.	.	.	.	.	.	.	.	.	.	.	.	.	.	.	.	.	.	.	.	.	.	.	.	.	.	.	.	.	.	.	.	.	.	.	.	.	.	.	.	.	.	.	.	.	.	.	.	.	.	.	.	100%
	4	.	.	.	.	.	.	.	.	.	.	.	.	.	.	.	.	.	.	.	.	.	.	.	.	.	.	.	.	.	.	.	.	.	.	.	.	.	.	.	.	.	.	.	.	.	.	.	.	.	.	.	.	100%
Sternotherus carinatus	NC_017607	.	.	.	.	.	.	.	.	.	.	.	.	.	.	.	.	.	.	.	.	T	.	.	.	.	.	.	.	.	.	.	.	.	.	.	.	.	.	G	.	.	.	.	.	.	.	.	.	.	.	.	.	96%
Sternotherus minor minor	1	.	.	T	.	.	.	.	.	.	.	.	.	.	.	.	.	.	.	.	A	T	.	.	.	.	.	A	.	.	.	.	.	.	.	.	.	.	.	.	.	.	.	.	.	.	.	.	.	.	.	.	.	92%
Sternotherus minor peltifer	1	.	.	T	.	.	.	.	.	.	.	.	.	.	.	.	.	.	.	.	.	T	.	.	.	.	.	.	A	.	A	.	.	.	.	.	.	.	.	.	.	.	.	.	.	.	.	.	.	.	.	.	.	92%
	2	.	.	T	.	.	.	.	.	.	.	.	.	.	.	.	.	.	.	.	.	T	.	.	.	.	.	.	A	.	A	.	.	.	.	.	.	.	.	.	.	.	.	.	.	.	.	.	.	.	.	.	.	92%
Sternotherus odoratus	1	.	.	.	.	.	.	.	.	.	T	.	.	.	.	.	.	.	.	.	A	T	.	.	T	.	.	A	.	.	.	.	.	.	.	.	.	.	.	G	.	.	.	.	.	.	A	.	.	.	.	.	.	87%

Real-time PCR assays of extractions (tissue-derived and filtered mesocosm water) were run with three replicates of each sample in the following 20µl mixes: 4.85µl of sterile water, 4µl of 5X GoTaq® Flexi Buffer (Promega), 0.4µl of 10mM dNTPs, 1.6µl of 25mM $MgCl_2$, 1µl of each 10µM primer, 0.15µl of GoTaq® Flexi DNA Polymerase, 1µl of 20X EvaGreen in water (Biotium), 2µl of 4µg/µl Bovine Serum Albumin (BSA), and 4µl of DNA extract. Mastercycler® ep *realplex* (Eppendorf) cycling conditions were as follows: an initial denaturation at 95°Celsius for 3 minutes; 40 cycles of denaturation at 95°Celsius for 30 seconds, annealing at 62°Celsius for 45 seconds, and extension at 72°Celsius for 1 minute; followed by a melting curve analysis transitioning from 60° to 95°Celsius over 20 minutes.

PrimerHunter

We loaded sequences into PrimerHunter version 1.0.1 for all genes sequenced, selecting target sequences versus non-target sequences. PrimerHunter searches for forward and reverse primers that combine with target sequences and discards primer candidates that combine with the non-targets. With all closely related congeners sequences for *Necturus alabamensis* and *Sternotherus depressus* included in the analysis we can ensure selected primers amplify our target species.
